# Achievements of the Cochrane Iran Associate Centre: Lessons Learned

**DOI:** 10.15171/ijhpm.2019.122

**Published:** 2019-12-11

**Authors:** Bita Mesgarpour, Sara Aghababa, Hamid Reza Baradaran, Payam Kabiri, Ali Kabir, Ahmad Sofi-Mahmudi, Ali Akbar Haghdoost

**Affiliations:** ^1^Cochrane Iran Associate Centre, National Institute for Medical research Development (NIMAD), Tehran, Iran.; ^2^Endocrine Research Center, Institute of Endocrinology and Metabolism, Iran University of Medical Sciences, Tehran, Iran.; ^3^Department of Epidemiology and Biostatistics, School of Public Health, Tehran University of Medical Sciences, Tehran, Iran.; ^4^Minimally Invasive Surgery Research Center, Iran University of Medical Sciences, Tehran, Iran.; ^5^Social Determinants of Health Research Centre, Institute for Futures Studies in Health, Kerman University of Medical Sciences, Kerman, Iran.

**Keywords:** Cochrane, Evidence-Based Practice, Iran, Systematic Review, Health Policy

## Abstract

Healthcare decision-making is a process that mainly depends on evidence and involves increasing numbers of stakeholders, including the consumers. Cochrane evidence responds to this challenge by identifying, appraising, integrating and synthesizing high-quality evidence. Recently, a collaborative effort has been initiated in Iran with Cochrane to establish a representative local entity. A variety of multifaceted interventions were conducted according to Cochrane’s strategy to 2020, such as producing evidence, making Cochrane evidence accessible, advocating for evidence and building an effective and sustainable organization. In this report, the authors present the two and half year performance and achievements of Cochrane Iran based on a comprehensive and systematic approach. This case might be an example of health diplomacy, which is initiated by a successful international collaboration and proceed with recognizing the importance of adherence to the strategic action plans and goals.

## Introduction

Systematic reviews are being increasingly employed by health policy-makers, researchers and even patients to assist decision-making processes.^1-4^

Cochrane is known as an independent non-for-profit organization that plays a crucial mediating role in creating, evaluating and disseminating reliable, invaluable and practical information in the format of systematic reviews and meta-analyses. With its well-deserved reputation, Cochrane has facilitated the use of applied research for practical purposes for more than two decades, including prevention, treatment and diagnosis.^5^ Cochrane potentially enables not only researchers to overcome low-value practices, but also other stakeholders to prohibit disinvestment decisions and assures them of the trustworthiness of its evidence.^6^

The global and independent Cochrane network has geographic groups in 43 countries that promote and support the use of Cochrane evidence in health policy-making and practice. These groups act as a regional focus for the activities of the Cochrane network and support Cochrane members and supporters through different approaches, including, but not limited to, the provision of training and the promotion of accessibility to the Cochrane library.^7^ The experiences of Cochrane UK,^8^ Switzerland,^9^ Poland,^10^ Russia,^11^ Sweden,^12^ and China,^13,14^ which are reflected in their annual reports, consist of informative lessons to learn and benchmarks.

Despite the small volume of systematic reviews in low- and middle-income countries,^15^ the efforts in Iran as a middle-income country have been focused on promoting advocacy for evidence-based practices and making high-quality evidences accessible in this geographical region.

A formal collaboration was therefore initiated with Cochrane. We encouraged our stakeholders to embark on the opportunities arising from this connection to Cochrane and made our performance a priority for the National Institute for Medical Research Development (NIMAD). This report describes the considerable achievements of Cochrane Iran Associate Centre over two and half years based on its strategic goals in line with Cochrane’s *Strategy to 2020*.

## Cochrane Iran as Part of the Global Cochrane Network

The idea of establishing Cochrane has been attributed to the British epidemiologist and medical doctor, Archibald Leman Cochrane, who made a ground-breaking contribution to the advocacy of randomized controlled trials in healthcare intervention research.^16,17^ The first systematic review in this field, entitled *Effective Care in Pregnancy and Childbirth,* was published 17 years later by Iain Chalmers, who was inspired by Cochrane.^18^ Eventually, the first Cochrane Centre was launched in 1993 in Oxford, UK.^19^ Currently, researchers, health professionals, patients, care providers and volunteers from more than 130 countries are involved in Cochrane as members (10 578) or supporters (61 099).^20^

To establish Cochrane Iran Associate Centre, NIMAD, as representative of the Iranian Ministry of Health, and Cochrane entered into a Memorandum of Understanding, dated December 2017. The Cochrane Iran, which is mostly a network rather than a centre, is located in Tehran within the NIMAD. This centre was officially launched by the Iranian Minister of Health and Medical Education and welcomed the message of Cochrane’s Chief Executive Officer on April 5, 2017.

## Achievements Based on Strategic Goals

**Figure 1:**
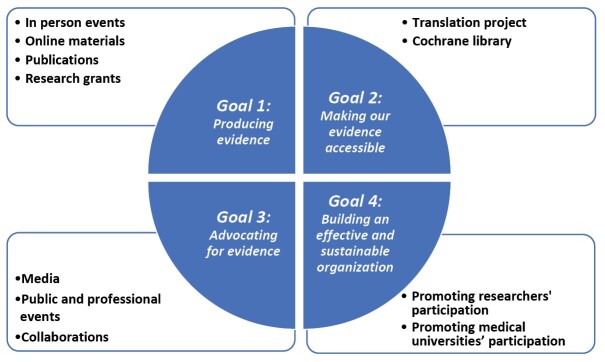
Cochrane Iran’s Strategy to 2020.

In line with Cochrane’s strategy to 2020,^2^ the activities for associate centres are organized around Cochrane’s four key goals: producing evidence, making our evidence accessible, advocating for evidence and building an effective and sustainable organization. The strategic plan of Cochrane Iran has been modified according to the existing regional gaps and its performance has been elaborated as follows, which is summarized in Figure 1.

### Goal 1. Producing Evidence

Cochrane Iran has promoted different types of Cochrane evidence, including Cochrane protocols and Cochrane reviews. This centre has also made efforts to build a bridge between high-quality evidence produced by Cochrane and the local stakeholders. Several national and international workshops were held, and access to the online learning resources of Cochrane served as an empowerment tool for the Cochrane authors.

#### In-Person Events

Cochrane Iran has facilitated face-to-face training opportunities for authors and researchers based on a systematic needs assessment at national and regional levels. In the last two and half years, Cochrane Iran organized 37 training courses around the country (in 10 sub-regions covering 64 medical universities). The titles of these workshops were extracted from the results of a national needs assessment and included “How to read a systematic review,” “How to develop a Cochrane protocol,” and “Basic level of evidence-based medicine.” A team of experts in the country coached these 2-3-day workshops; however, the course plan and training materials were provided by Cochrane Iran. Candidate registration and course evaluation were part of Cochrane Iran’s job, so as to make sure the standards were met.

#### Online Materials

Cochrane Iran has been distributing Cochrane’s online learning events, including webinars regularly held to raise awareness and offer opportunities to authors in Iran. The recorded webinars are published on a local video-sharing website entitled Aparat, because of limited access to YouTube in Iran.

#### Publications

*Book:* Cochrane Iran contributed to and supported the update of the best-selling Persian book entitled “*Systematic review and meta-analysis* ,” published in 2019, which is one of the reference books for post-graduate students now.^22^

*Cochrane Protocols and Reviews:* Cochrane Iran is promoting production of Cochrane evidence by Iranian researchers and monitoring the performance. Forty-eight Cochrane reviews and 33 Cochrane protocols with at least one author affiliated to Iran have been identified from 2004 to 2019. Since Cochrane reviews should be updated, there are several versions for each review, reaching a total of 110 publications. Nonetheless, seven reviews and seven protocols were withdrawn. The first Cochrane review protocol developed by Iranian authors was published in January 2005^23^ and the first full systematic review appeared in the Cochrane library in January 2007.^24^

*Paper:* Thirteen Persian papers have been translated or their data extracted and evaluated for risk of bias and to ensure that they conform to the requirements of Cochrane review authors. These requests have been received by email or through the portal TaskExchange.

#### Research Grants

NIMAD introduced a top-paper grant in May 2018 for the first/corresponding author of papers published in Q1 or journals with IF = 6 or more, and the Cochrane Database of Systematic Reviews and Iranian authors would be the potential applicants of this grant. Receiving support for developing Cochrane review for Iranian authors who published their protocol up to one year ago has been added to the criteria of NIMAD Elite Grants in February 2018. These changes occurred following Cochrane Iran’s suggestion to NIMAD to hold several initiatives for supporting the production of Cochrane reviews by Iranian researchers.

### Goal 2: Making Our Evidence Accessible

In order to achieve this goal, Cochrane Iran has taken the responsibility of translating the Cochrane content into Persian. Although healthcare professionals are often capable of understanding English content, most of the other stakeholders, including patients, policy-makers and the media, require translated documents. Cochrane Iran supports access to the Cochrane library – the major product of Cochrane.

#### Translation Project

**Figure 2:**
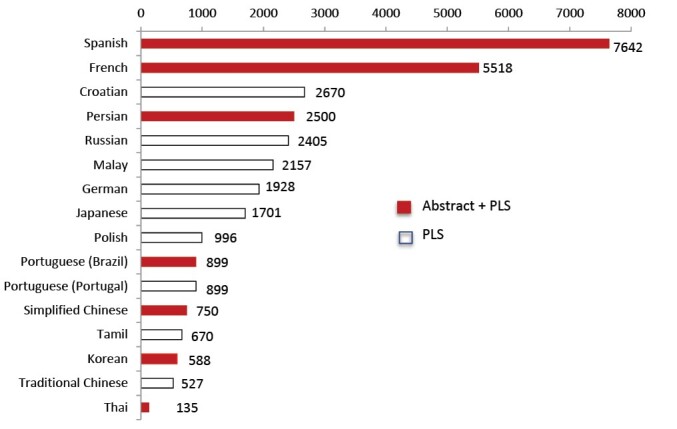
The Number of Translated Cochrane Abstracts and PLS Per Language, October 1, 2019. Abbreviation: PLS, Plain Language Summaries.

Cochrane Iran had translated 2500 Cochrane abstracts into Persian by October 1, 2019. To achieve this figure, a translating team was made up of ten graduate medical students familiar with medical science issues and fluent in English. The translations of Cochrane abstracts into Persian consist of scientific abstracts and Cochrane Plain Language Summaries (PLS), which are targeted for the general population. Persian is the third language of translated Cochrane abstracts, including PLS, in terms of the sheer number of translated abstracts (Figure 2). The numbers of translation vary across the languages because of different reasons such as earlier inception, financial resources and the capacity of dependent Cochrane centre(s).

These Persian abstracts have been published in the Persian Cochrane library on www.cochrane.ir and had been viewed more than two million times by March 2019. Table 1 presents the most viewed abstracts.

**Table 1:** The Top 10 Most Viewed Persian Cochrane Abstracts on Cochrane.ir, October 1, 2019

**Rank**	**Title of Cochrane Review**	**DOI**	**Views**
1	Betahistine for symptoms of vertigo	10.1002/14651858.CD010696.pub2	241 952
2	Pregabalin for pain in fibromyalgia in adults	10.1002/14651858.CD011790.pub2	117 607
3	Gabapentin for fibromyalgia pain in adults	10.1002/14651858.CD012188.pub2	73 931
4	Aripiprazole for autism spectrum disorders (ASD)	10.1002/14651858.CD009043.pub3	62 699
5	Early versus delayed postoperative bathing or showering to prevent wound complications	10.1002/14651858.CD010075.pub3	55 293
6	Blood CEA levels for detecting recurrent colorectal cancer	10.1002/14651858.CD011134.pub2	45 743
7	*Ganoderma lucidum* (Reishi mushroom) for cancer treatment	10.1002/14651858.CD007731.pub3	40 549
8	D-dimer test for excluding the diagnosis of pulmonary embolism	10.1002/14651858.CD010864.pub2	39 677
9	Antidepressants and benzodiazepines for panic disorder in adults	10.1002/14651858.CD011567.pub2	29 740
10	Topical antibiotics for preventing surgical site infection in wounds healing by primary intention	10.1002/14651858.CD011426.pub2	24 531

Abbreviation: CEA, carcinoembryonic antigen.

Cochrane Iran has engaged 21 volunteer translators up to now, who are mainly students of medical universities. To date, one of these translators has been interviewed as part of *Cochrane’s 30 under 30* series. Furthermore, nine podcasts about popular Cochrane reviews have been translated and recorded by these volunteers. One of these podcasts, which was about Betahistin for the treatment of vertigo, has been ranked among the top viewed Cochrane podcasts of 2018, with more than 16 000 views. An interesting point is that the abstract corresponding to this podcast is also the top viewed Persian abstract (Table 1). The analysis of these views showed that most of them have been directed from a Google search. The Persian abstracts and podcasts have been disseminated through domestic channels, such as the social media.

Furthermore, Persian has been added to the languages of Cochrane.org on October 1, 2019 and Cochrane website is now being translated into 15 languages.

#### Cochrane Library

Cochrane Iran has supported maintaining access to the Cochrane library for all medical universities and affiliated centres and teaching hospitals through the Ovid platform by way of a national subscription. Our collaborative effort has enhanced the access and utilization of the Cochrane library. We compared the top 40 out of 236 countries with the most frequent visits to the Cochrane library. Although Table 1 depicts a gentle decrease in the ranking of Iran, the number of visits implies a growth from 44 171 to 50 340 in 2017 and 2018 (Table 2).The numbers, however, might have been underreported, since a considerable number of Iranians use virtual private networks to unblock restricted access to websites, which augmented after the 2018 US sanctions.

**Table 2:** Visits to the Cochrane Library by Country in 2017 and 2018

**Country**	**Visits (2017)**	**Country**	**Visits (2018)**
United States	1 659 507	United States	2 116 429
United Kingdom	1 029 729	United Kingdom	1 301 034
Australia	542 378	Australia	671 740
Canada	286 378	Canada	356 171
India	222 172	India	317 507
Brazil	187 690	China	269 073
China	177 273	Spain	258 720
The Netherlands	153 125	Brazil	256 695
Spain	153 074	Germany	191 251
Germany	150 974	The Netherlands	184 920
Italy	134 235	Italy	165 316
Japan	111 039	Taiwan	154 832
Taiwan	107 564	Japan	152 975
France	95 615	Mexico	149 869
Mexico	90 355	France	123 512
Indonesia	77 410	Indonesia	99 161
New Zealand	74 464	Ireland	92 872
Ireland	73 252	Turkey	87 132
Thailand	68 299	New Zealand	86 984
Norway	66 662	Chile	83 167
Turkey	66 070	Thailand	82 279
Sweden	63 642	Norway	81 192
Belgium	61 049	Colombia	81 038
Malaysia	57 809	Malaysia	79 630
Switzerland	57 379	Sweden	78 377
South Korea	57 242	Belgium	72 976
Colombia	56 805	Philippines	70 890
Chile	54 068	Switzerland	70 139
Philippines	48 800	South Korea	67 402
Egypt	46 425	Singapore	65 062
South Africa	45 262	Egypt	61 904
Denmark	44 391	Russian Federation	61 688
Singapore	44 207	Argentina	60 566
**Iran**	**44 171**	Denmark	57 407
Russian Federation	41 126	South Africa	53 827
Portugal	37 604	Peru	52 775
Saudi Arabia	36 116	**Iran**	**50 340**
Argentina	35 859	Hong Kong	50 021
Hong Kong	34 914	Portugal	46 961
Peru	34 084	Saudi Arabia	46 592

### Goal 3: Advocating for Evidence

Our team has promoted Cochrane and its work in Iran through the 10-subregional medical universities, which represent all 64 Iranian medical universities/schools. In 2017, after introducing Cochrane Iran Associate Centre to Iranian medical universities, medical scholars and students were recommended to seek collaboration with Cochrane Iran. We also cooperated to promote the contribution of local researchers in joining the working groups of Iranian SYstematic REview Network (ISYREN) as an ancillary network of Cochrane Iran.

#### Media

The media plays a pivotal role in communication with people and presents a window of opportunity to researchers to face a broad general audience in the community. Cochrane Iran embraces this potential value in order to present Cochrane products. The following types of media represent our efforts to provide scientific news, information and evidence for the public.

*Newspapers:* We have published two feature articles in Salamat newspaper – weekly newspaper in healthcare – entitled “Systematic review of the benefits of Yoga for health: What do researchers say about Yoga?” and “Cochrane recommendations on postoperative knowledge: Three points to follow after surgery.” These features depicted Cochrane evidence in a plain language for the public with a description of Cochrane Iran and its Persian library.

*Webpage:* We created iran.cochrane.org as a sub-domain of cochrane.org and cochrane.ir as a local host specified for the dissemination of translated abstracts (Iranian Cochrane Library). In its local host, this website might increase the retrieval of Persian Cochrane abstracts for people searching Persian words in search engines such as Google. The most viewed abstracts are retrieved in the top ten Google records following the search of particular medication names. The traffic analysis of cochrane.ir indicates that it has more than 3000 visits per day. The traffic analysis of cochrane.ir indicates that it has more than 5000 visits per day. Furthermore, 723 pages from 74 websites have a link to 295 target pages on this website, making the website rank much more improved.

*Social media:* Popular social media networks, such as Telegram, Instagram, Twitter, and Aparat were used to introduce Cochrane Iran to the local community (Table 3).

**Table 3:** Cochrane Iran Statistics in the Social Media, October 1, 2019

**Telegram Channel:**118 posts, 886 subscribers, 161 851 views
**Aparat Channel:**127 videos, 13 followers, 15 265 views
**Instagram:**59 posts, 254 followers
**Twitter:**103 posts, 148 followers

#### Public and Professional Events

Cochrane Iran has been introduced to health and medical researchers from May 2017 to October 2019 in more than 21 scientific events, including workshops, seminars and conferences. Moreover, Cochrane Iran has put tremendous effort into the translation of the book of *Testing Treatments* , which promotes the understanding and evaluation of health treatment by the public and patients. The website called Testing Treatments Interactive, which has been translated into 14 languages, provides a valuable resource for developing critical thinking in treatment claims. The launch plan of the Persian website accompanies the launch of the Persian translation of the book *Testing Treatments* in the early 2020.

#### Collaborations

Involving consumer’s and health professionals’ associations in advocating for evidence has been defined as a goal in Cochrane Iran’s strategic plan. An agreement was reached with the Medical Council of the Islamic Republic of Iran in June 2019 and a meeting has so far been planned with the Commission of Medical Scientific Associations.

### Goal 4: Building an Effective and Sustainable Organization

Cochrane Iran endeavours to be a diverse, inclusive and transparent that provides all Cochrane citizen scientists with adequate enthusiasm and skills an opportunity to support this national organization. This centre is guided by the Cochrane principles, governed accountably and managed efficiently and strives for the optimal use of its resources.

#### Promoting Researchers’ Participation

**Figure 3:**
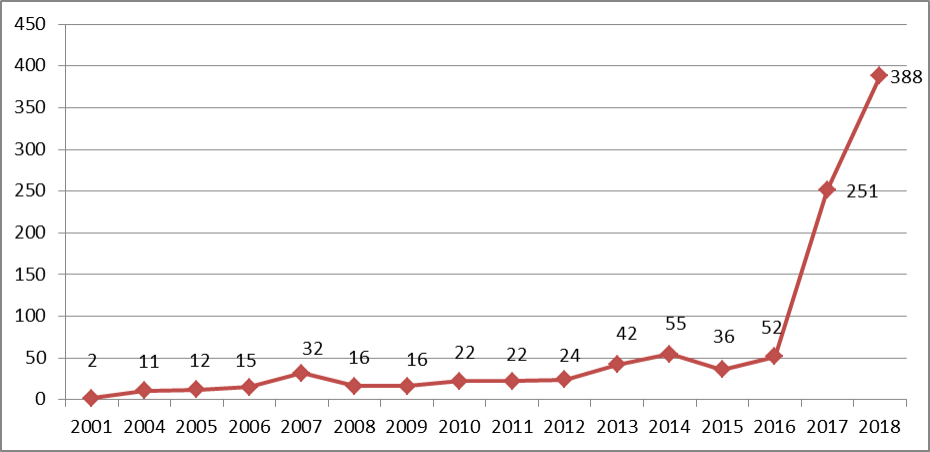
The Trend of Researchers With an Iranian Affiliation Who Are Contributing to Cochrane.

The analysis of the characteristics of 996 Cochrane members who were affiliated to an Iranian university/institute showed a significant increase in the number of registered members or supporters in the recent years, from 52 in 2016 to 251 and 388 in 2017 and 2018, respectively (Figure 3). This growth is mostly due to Cochrane’s new membership scheme, ie, the Membership Project that launched in 2017 (n = 618, 62%).

**Figure 4:**
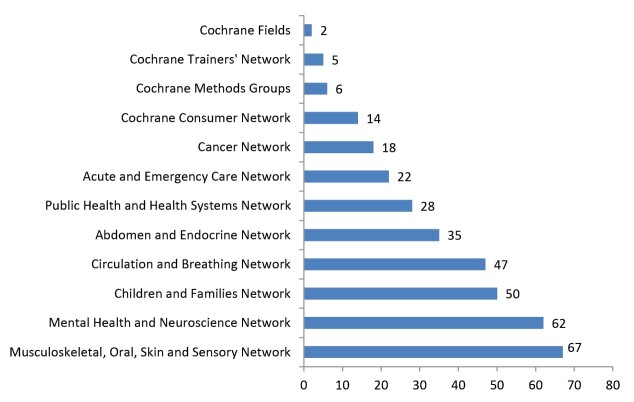
The Number of Iranian Researchers With a Role in Cochrane Review Networks, Method Groups and Fields.

Figure 4 illustrates the primary groups of Cochrane’s Iranian members, which were classified into one out of eight Cochrane networks. This figure shows that 329 (33%) of the members have joined in a Review Group, 14 in the Consumer Network, five in Trainers’ Networks, six in Methods Groups and two in Cochrane Fields. Iranian members play a part in 48 out of the 52 Cochrane Review Groups. The “*Schizophrenia Group,* ” “*Heart Group,* ” “*Back and Neck Group,* ” and “*Cystic Fibrosis and Genetic Disorders Group* ” have the highest number of members (37, 22, 16, and 16, respectively). The five methods groups include“*Equity Methods Group* ” with two members and the “*Adverse Effects Methods Group,* ” “*Economics Methods Group,* ” “*Editorial and Methods Department* ” and “*Information Retrieval Methods Group.* ” The two Cochrane fields include “*Cochrane Child Health* ” and “*Cochrane Rehabilitation.* ”

#### Promoting Medical Universities’ Participation

Iranian medical universities have been encouraged to build an internal network of contributors to systematic reviews (ISYREN) in the following groups and define their strengths and gaps.

- Review methods
- Search and information retrieval
- Knowledge translation and communicating evidence
- Data extraction
- Quality assessment
- Statistical methods

Iran University of Medical Sciences, Tehran, Iran and Shahid Beheshti University of Medical Sciences, Tehran, Iran have initiated their network. Kermanshah University of Medical Sciences, Kermanshah, Iran is working on establishing a research centre on systematic reviews. Nevertheless, the lack of human and institutional capacity-building affects the outcome of these university networks.

## Conclusion

The present report aimed to describe and highlight the two-year achievements of Cochrane Iran according to the predefined goals. Cochrane Iran has been using a comprehensive approach to promote the valiant attempts of Iranian researchers with sustained efforts to produce and implement high-quality evidence under Cochrane’s leadership. The experience of NIMAD to building up a branch of an international organization in Iran has been relatively successful. The key for effective collaboration with the Cochrane network might be developing a strategic plan and being committed to the goals and values of the organization. NIMAD also provided an opportunity for Cochrane Iran to network with universities academic staff and present its program in several events. Furthermore, producing Cochrane abstracts in Persian might have increased access to the high-quality health evidence by healthcare providers, professionals, and workers as well as patients and family carers. Having support from a national granting body to initiate Cochrane Iran activities, cooperation with Ministry of Health, building a network with medical universities/research centres, holding workshops for different target groups, being involve in updating a national reference book in Persian, and being inclusive in collaboration with all interested individuals were fruitful lessons, which can be learned from our experiences. The efforts of the centre, based on the four goals could reflect Cochrane knowledge translation of goals in Iran. However, to align with Cochrane Strategy 2020 and to put Cochrane evidence at the heart of health decision-making in Iran, our team has yet a lot more to work on. Cochrane Iran can be strengthened through the following steps:

- Supporting health policy-makers to use Cochrane evidence
- Making Persian abstracts of Cochrane reviews more accessible to the decision-makers
- Improving advocacy in the social media
- Promoting the grading quality of evidence and the strength of the recommendations, including guidelines provided by the national health technology assessment entities
- Building the capacity in Cochrane Iran volunteers to engage more in knowledge translation activities.

## Acknowledgements

Cochrane Iran would like to express its gratitude to Prof. Reza Malekzadeh (Director of NIMAD) and Prof. Shahin Akhondzadeh (Vice-Chancellor of NIMAD), Dr. Mona Nasser (former Board Member [Trustee] of Cochrane), Dr. Shadi Kolahdoozan (Translation Team Manager of the Persian Cochrane library), the team of Cochrane’s Chief Executive Officer, especially Mark Wilson, Sylvia de Haan, Lucie Binder, and Lorna McAlley as well as the team of Cochrane Knowledge Translation, especially Juliane Ried, Muriah Umoquit, and Jo Anthony, for their support. This study has been partially funded by NIMAD under contract No. 962656.

## Ethical issues

Not applicable.

## Competing interests

Authors declare that they have no competing interests.

## Authors’ contributions

BM has conducted acquisition, analysis and interpretation of data and statistical analysis. SA has involved in conception and design and drafting the manuscript. HRB drafted of the manuscript and obtained funding. PK has involved with critical revision of the manuscript for important intellectual content, statistical analysis and provided technical, support. AK critically revised the manuscript and provided material support. ASM has involved in providing statistics and critical revision of the manuscript. AAH supervised the manuscript and provided administrative support.

## Authors’ affiliations

^1^Cochrane Iran Associate Centre, National Institute for Medical research Development (NIMAD), Tehran, Iran. ^2^Endocrine Research Center, Institute of Endocrinology and Metabolism, Iran University of Medical Sciences, Tehran, Iran. ^3^Department of Epidemiology and Biostatistics, School of Public Health, Tehran University of Medical Sciences, Tehran, Iran. ^4^Minimally Invasive Surgery Research Center, Iran University of Medical Sciences, Tehran, Iran. ^5^Social Determinants of Health Research Centre, Institute for Futures Studies in Health, Kerman University of Medical Sciences, Kerman, Iran.
